# Improving Blood Pressure Among African Americans With Hypertension Using a Mobile Health Approach (the MI-BP App): Protocol for a Randomized Controlled Trial

**DOI:** 10.2196/12601

**Published:** 2019-01-25

**Authors:** Lorraine R Buis, Katee Dawood, Reema Kadri, Rachelle Dawood, Caroline R Richardson, Zora Djuric, Ananda Sen, Melissa Plegue, David Hutton, Aaron Brody, Candace D McNaughton, Robert D Brook, Phillip Levy

**Affiliations:** 1 Department of Family Medicine University of Michigan Ann Arbor, MI United States; 2 Integrative Biosciences Center Department of Emergency Medicine Wayne State University Detroit, MI United States; 3 School of Public Health University of Michigan Ann Arbor, MI United States; 4 Department of Emergency Medicine Vanderbilt University Medical Center Nashville, TN United States; 5 Division of Cardiovascular Medicine Department of Internal Medicine University of Michigan Ann Arbor, MI United States

**Keywords:** hypertension, mHealth, blood pressure, smartphone, mobile phone

## Abstract

**Background:**

African Americans shoulder significant disparities related to hypertension (HTN), which is a serious public health problem in the city of Detroit, Michigan, where more than 80% of the population is African American. Connectivity through smartphones, use of home blood pressure (BP) monitoring, and newly developed mobile health (mHealth) interventions can facilitate behavioral changes and may improve long-term self-care for chronic conditions, but implementation of a combined approach utilizing these methods has not been tested among African American patients with uncontrolled HTN. Since African Americans are more likely than other racial or ethnic subgroups to utilize the emergency department (ED) for ambulatory care, this presents an opportunity to intervene on a population that is otherwise difficult to reach.

**Objective:**

The MI-BP app aims to reduce health disparities related to HTN in the community by employing a user-centered intervention focused on self-BP monitoring, physical activity, reduced sodium intake, and medication adherence. We seek to test the efficacy of MI-BP, an mHealth app for HTN self-management, on BP control (primary aim), physical activity, sodium intake, and medication adherence (secondary aim) in African Americans with HTN. This study also seeks to evaluate the cost-effectiveness of MI-BP when compared with usual care methods.

**Methods:**

This is a 1-year randomized controlled trial that will recruit individuals who have uncontrolled HTN from 2 EDs in the city of Detroit, with a planned sample size of 396 randomized participants. To be eligible for inclusion, potential participants must be African American, 25 to 70 years old, previously diagnosed with HTN, have a smartphone compatible with MI-BP, and have uncontrolled BP at triage and on repeat measurement at least 1-hour post triage vitals. Once a participant is deemed eligible, all study procedures and subsequent follow-up visits (8 in total) are conducted at the Wayne State University Clinical Research Service Center. We seek to determine the effect of MI-BP on BP for 1 year (using BP control and mean systolic BP as coprimary outcomes and physical activity, sodium intake, and medication adherence as secondary outcomes) compared with usual care controls.

**Results:**

Recruitment for this study began in January 2018. The study will continue through 2021.

**Conclusions:**

As the first of its kind conducted in an ED setting, MI-BP was designed to document the efficacy and acceptability of a multicomponent mHealth approach to help African Americans with uncontrolled BP modify their lifestyle to better manage their HTN. We expect to lay the foundation to sustainably reduce HTN-related health disparities through better integration of multiple behavior self-monitoring and improve outcomes for those who traditionally rely on the ED for chronic disease care.

**Trial Registration:**

ClinicalTrials.gov NCT02360293; http://clinicaltrials.gov/ct2/show/NCT02360293

**International Registered Report Identifier (IRRID):**

RR1-10.2196/12601

## Introduction

### Background

Hypertension (HTN) is one of the most important cardiovascular disease risk factors affecting more than 100 million Americans under the new American College of Cardiology/American Heart Association (AHA) guidelines [1,2]. However, only about half of those with HTN achieve blood pressure (BP) control, and about 15.9% remain unaware of their condition [3]. Compared with whites, African Americans are more likely to develop HTN and have uncontrolled BP [4], increasing the risk of premature cardiovascular morbidity and mortality. African Americans are also more likely to utilize the emergency department (ED) for ambulatory care of chronic conditions such as HTN [5], a factor strongly linked with adverse cardiovascular events and diminished awareness of HTN [6], as well as lower BP control [7]. Although there are many reasons for such patterns of ED utilization, including poor access to primary care, and the ability to receive care at all hours regardless of the ability to pay, it serves to highlight the challenges certain populations face, which may have downstream effects on self-management.

Recommendations for improving HTN-related outcomes have been consistent for decades: maintain a healthy weight, reduce daily sodium intake, increase physical activity, and comply with antihypertensive therapy, as prescribed [8]. Despite strong evidence supporting these recommendations, facilitating the necessary behavior changes in patients with HTN remains difficult. African Americans, in particular, are less likely than whites to report adherence with such lifestyle and behavioral changes [3]. Moreover, existing interventions for improving BP typically focus on targeting 1 behavior, which may not be sufficient for improving BP control [9-12]. However, comprehensive evaluations of multiple health behavior change interventions, especially as they relate to BP control among African Americans, are lacking from the literature.

Accordingly, we sought to develop and test a mobile health (mHealth) approach to deliver a multiple behavior change intervention targeting BP reduction in African American ED patients with uncontrolled HTN. About 95% of American adults own some form of a mobile phone, and smartphone adoption is about the same in white and African American populations (77% and 75%, respectively) [13], yet African Americans are more dependent on smartphones for internet access than whites (24% vs 14%, respectively) [14], suggesting that an mHealth approach may be particularly useful in this population. Since BP is routinely measured in EDs, visits to the ED provide a unique opportunity to both identify patients with uncontrolled HTN and intercede, particularly in African American communities where regular interaction with the health care system may be lacking.

### Study Objective

The purpose of this study is to establish the efficacy of MI-BP, a multiple behavior change intervention delivered via mHealth that supports achieving and maintaining BP control in African Americans who have uncontrolled HTN and are recruited from 2 urban EDs. This study also seeks to evaluate the cost-effectiveness of MI-BP compared with enhanced usual care methods.

Our primary study aim is to determine the effect of MI-BP on BP for 1 year (using BP control and mean systolic blood pressure [SBP] as coprimary outcomes) compared with usual care controls, in a 1-year randomized controlled trial (RCT) (NCT02955537). We hypothesize that at 1 year, BP control rates will be significantly greater in the MI-BP arm than in usual care. We also hypothesize that at 1 year, mean SBP will be significantly lower in the MI-BP arm than in usual care. Our second aim is to determine the effect of MI-BP on secondary outcomes (physical activity, sodium intake, and medication adherence) compared with usual care controls, in a 1-year RCT. We hypothesize that at 1 year, measures of physical activity, sodium intake, and medication adherence will be significantly better in the MI-BP arm than in usual care. Our third aim is to evaluate the cost-effectiveness of MI-BP compared with usual care methods. We hypothesize that the MI-BP approach will provide good value for money, both within-trial and long term. Here we describe the MI-BP approach that was developed for this study.

## Methods

### Overview

This is a 1-year, 2-arm, RCT of MI-BP versus enhanced usual care plus follow-up. The methods for this study have been approved by Wayne State University’s (WSU) Institutional Review Board (IRB#: 040416M1F) and the University of Michigan IRBMED (HUM00114202).

### MI-BP Intervention Description

MI-BP is a comprehensive, user-centered, multicomponent intervention that targets multiple behavior changes for managing HTN. The MI-BP intervention was developed building on our previous work with the BPMED text message intervention to improve HTN medication adherence in the same population [15]. Our design process incorporated target end user feedback from the Hypertension Community Advisory Board in Detroit, Michigan.

The MI-BP intervention includes a smartphone-based app that incorporates the following components, which users are encouraged but not required to use. Vibrent Health (Fairfax, VA), a digital health company, was engaged to develop the app, online portal, and server platforms necessary to support this project.

#### Home Blood Pressure Monitoring and Tracking

**Figure 1:**
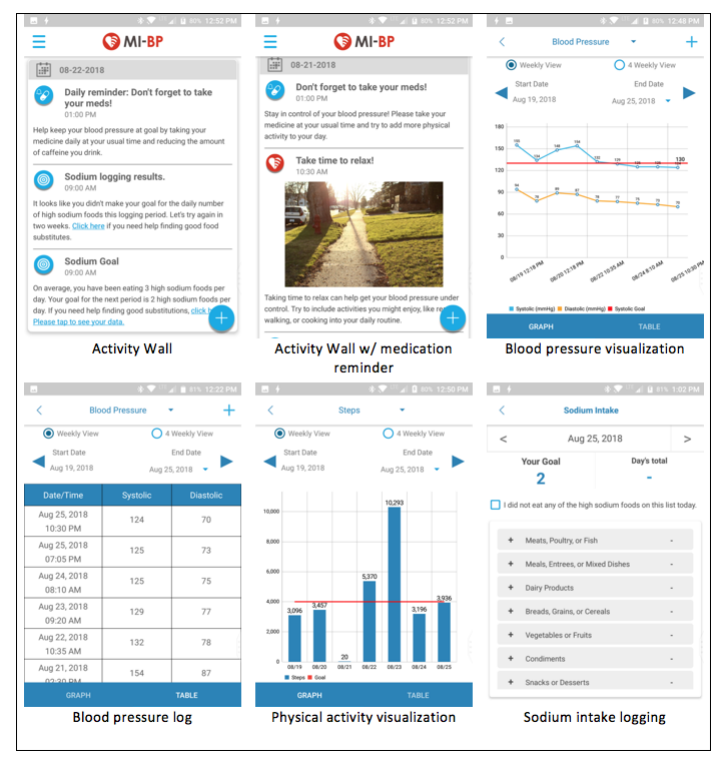
Screenshots from the MI-BP app.

MI-BP allows users to revicew both 1- and 4-week graphs of BP readings, collected via study-issued home BP cuffs. In addition to graphs, numerical logs of 1- and 4-week BP data are also available for review (see Figure 1 for screenshots of the MI-BP app). BP data can be either digitally synced, or manually entered into the MI-BP app. Participants with arm circumferences of 23 to 45 cm, which represents the majority of users, will receive a Bluetooth-enabled digital BP monitor (A&D UA-651BLE) that can store up to 30 BP measurements. With a touch of a button, this cuff can automatically sync collected measurements with the MI-BP app. For the participants who have an arm circumference between 42 and 60 cm, we will provide an extra-large arm monitor (LifeSource A&D UA-789). These cuffs are not Bluetooth-enabled and require manual data entry.

Users are instructed to measure and sync (or manually enter) their BP to the MI-BP app, at home, for a minimum of at least 3 days per week; however, daily self-monitoring and syncing are encouraged. When taking BP at home, users are instructed to take 3 consecutive readings and adhere to the following guidelines:

- Relieve yourself in the bathroom before taking BP, if needed.
- Keep arm at heart level by resting it on a table during monitoring.
- Sit in a chair with a back and with feet flat on the floor for at least 5 min before taking BP.
- Avoid tobacco, caffeine, or alcohol for 30 min before taking BP.
- Avoid taking BP right after exercise, when emotionally upset, or in pain.
- Avoid talking while taking BP.

In the event that a participant records a BP with systolic reading of greater than 180 mmHg or less than 100 mmHg, or a diastolic reading of greater than 110 mmHg, the MI-BP users are then instructed by the study staff at baseline, as well as by automated notifications within the app at the time of the elevated reading, to do the following:

- Wait for 5 min and then check BP again.
- If the SBP is still above 180 mmHg or less than 100 mmHg, or if the diastolic BP is still above 110 mmHg for 3 days in a row, call the research staff.
- Report to the ED and follow up after with a call to the research staff if experiencing symptoms of dizziness, chest pain, severe headache, visual changes, or numbness or weakness in face or extremities.

#### Physical Activity Monitoring and Tracking

MI-BP also allows users to view numerical logs, as well as 1- and 4-week graphs of physical activity data, such as steps counts and miles collected via study-issued Fitbit Zip pedometers (see Figure 1 for screenshots of the MI-BP app). These devices can store up to 30 days of step-count data. Recent work by Tully et al found the Fitbit Zip to be a valid measure of physical activity in free-living adults [16]. Users are instructed to wear their Fitbit daily and to sync the device at least once per week.

#### Sodium Intake Monitoring and Tracking

In addition to BP and physical activity, MI-BP allows users to track their daily intake of high-sodium foods using a checklist approach and monitor their intake over time with 1- and 4-week graphs. Rather than asking users to self-report all of the food they eat in a day, we take a more adaptive approach to estimate sodium intake. The MI-BP users are asked to self-monitor their intake of high-sodium foods, using a checklist-type log available within MI-BP. Our checklist comprises 7 categories, with 3 to 8 items per category. The categories and items included within were based on resources from the University of California, San Francisco [17], Centers for Disease Control and Prevention [18], and work by Smith et al [19], and they were refined based on our own team’s expertise working within this target population. Each time an MI-BP user eats one of the foods on the checklist (regardless of portion size), we ask the participants to indicate this within the sodium log. At the end of the day, users should have a count of the number of times a high-sodium food was consumed in the day. Although we encourage users to track their intake of high-sodium foods daily, this is not required (see Figure 1 for screenshots of the MI-BP app).

#### Goal Setting

Physical activity goal setting is conducted weekly and step-count goals are displayed within the MI-BP app, as well as sent to users via push notification. Step-count goals are gradually incremented based on previous work from our team [20-24] and are calculated based on an average of 7 consecutive days of data, during which at least 5 of the days must be valid (ie, more than 200 steps per day). Calculated goals are never more than 600 steps more than the previous goal, which allows goals to be gradually incremented in order to reduce adverse events.

Sodium intake goal setting is conducted every 2 to 4 weeks, and sodium intake goals are displayed within the MI-BP app, as well as sent to users via push notification (see Figure 1 for screenshots of the MI-BP app). MI-BP users are instructed to intensively self-monitor their diet every day for the first week of their intervention period. This 1-week period serves 2 purposes: (1) as an acclimation period in which the participants become familiar with the act of tracking daily sodium intake and (2) for use as a baseline assessment from which an average daily sodium intake measure is calculated and the first daily sodium goal is issued. After this baseline monitoring period, users are asked to log their intake of high-sodium foods for a 3-day period approximately 2 weeks later. If the user meets their sodium goal (defined as submitting log data for all 3 logging days where all 3 values are less than or equal to the goal), then the user will be issued a new, lower goal based on the submitted data and will be asked to again intensively monitor their food intake 4 weeks later. Failure to meet the sodium goal during the logging period will prompt the system to ask the user to complete another 3-day logging period 2 weeks later. The sodium goals are calculated using the following algorithm:

- For those who log an average of 2 to 6 items per day during their logging period, their new goal is 1 item less than the average.
- For those who log an average of 7 to 12 items per day during their logging period, their new goal is 2 items less than the average.
- For those who log an average of 13+ items per day during their logging period, their new goal is 3 items less than the average.

#### Messaging

MI-BP provides users with 4 different types of messages, which are sent via push notification and in-app messaging. These messages include educational messaging, motivational messaging, tailored messaging, and daily medication reminders. In addition to the daily medication reminders, MI-BP sends about 7 messages per week. To enhance long-term engagement with the intervention from participants, message content, frequency, and timing are varied and tailored wherever possible, to maximize user engagement (see Multimedia Appendix 1 for sample messages and Figure 1 for screenshots of the MI-BP app).

##### Educational Messaging

MI-BP provides users with educational messaging via push notification and in-app messages. Educational messaging topics include tips pertaining to tracking BP, physical activity, and sodium; tips pertaining to increasing physical activity, improving diet, increasing medication adherence, lowering BP, and making or maintaining behavior changes; and tips pertaining to overcoming barriers to behavior changes.

##### Motivational Messaging

In addition to educational messaging, motivational messaging and words of encouragement to meet goals are also provided to MI-BP users.

##### Tailored Messaging Relevant to Individual Participants

MI-BP also provides tailored messaging to users, including tips for overcoming specific, self-reported barriers to behavior changes (assessed at baseline and repeated at 6 months) and previous history of app usage (eg, reminders to sync or log data), along with reminders about self-monitoring protocols, and support for goal attainment, where applicable. For example, positive reinforcement messages are sent to the participants when goals are met, and action-oriented messages promoting behavior changes are sent when goals are not met.

##### Medication Reminders

MI-BP also enables users to set up daily medication adherence reminders (see Figure 1 for screenshots of the MI-BP app). The number of messages and the timing of those messages are customizable by the user to account for multiple antihypertensive medications and multiple doses per day. Although the study staff help users set up reminders at the time of randomization, users are free to modify these reminders at any time.

### Clinical Setting

Participant recruitment occurs at the Detroit Medical Center (DMC) in the EDs of Detroit Receiving Hospital (DRH) and Sinai-Grace Hospital (SGH), both located in Detroit, Michigan. In Detroit, where 59% of the population lives in a medically underserved area, and an equal number (32.5% of individuals and 27% of families) live in poverty [25], reliance on the ED for primary care of chronic conditions such as HTN is commonplace. Once enrolled, the study participants complete all subsequent visits on the campus of WSU, in the Clinical Research Service Center (CRSC).

#### Recruitment

Recruitment occurs on site in the 2 EDs with active screening 24 hours per day, 7 days per week, 365 days per year. All patients that enter the ED are screened by trained research staff through the DMC electronic medical records system (Citrix Systems, Inc). Once a patient is deemed eligible, a research staff member approaches the patient’s treating physician and asks if this study will benefit the patient’s medical needs. Upon agreeance of the physician, the patient is then approached and educated on the study. If the patient is interested and agrees to participate in the study, an informed consent form is signed by the patient before any study procedure is done. Participants recruited to the MI-BP study are also approached during their baseline appointment to participate in an optional biorepository of blood samples collected during the trial. The biorepository has a separate consent process, which states that the participants’ data from MI-BP are associated with their blood sample.

### Eligibility Criteria

#### Inclusion Criteria

To be enrolled, potential participants must be African American and between the ages of 25 to 70 years, have been previously diagnosed with HTN, have a smartphone compatible with the mobile intervention, and have uncontrolled BP (SBP>135 mmHg) at triage and on repeat measurement using the BpTRU (Smiths Medical PM Inc, Waukesha, WI) device at least 1-hour post triage vitals.

#### Exclusion Criteria

Individuals who are pregnant; have serious existing medical conditions that may make BP control difficult or necessitate frequent hospitalization (ie, previous diagnosis of resistant HTN, steroid-dependent asthma or emphysema, cirrhosis or hepatic failure, stage C or D chronic heart failure, stage IV or V chronic kidney disease, and terminal cancer or ongoing active chemotherapeutic or radiation therapy); have a history of other serious medical conditions (eg, stroke, dementia, myocardial infarction or known coronary artery disease); or have a history of alcohol or drug abuse as determined by the CAGE-AID-Cut down, Annoyed, Guilty, Eye-opener Adapted to Include Drugs questionnaire (excluded if 2 or more) [26] are excluded.

Those who meet the eligibility criteria are enrolled in the ED and given a subsequent appointment for follow-up 1 to 2 weeks later at the WSU CRSC where BP is remeasured using the BpTRU device. To ensure that we are indeed including a sample with uncontrolled HTN, the participants who have a SBP<130 mmHg at that time are deemed ineligible and excluded from the study.

### Sample Size

On the basis of data from our previous studies, we estimate that 30% of usual-care participants will achieve BP control (BP<130/80 mmHg) by 1 year. We consider at least 17.5% higher control rates for the intervention arm to be clinically meaningful. On the basis of a 2-sided chi-square test of comparing 2 proportions at a 5% level of significance, the power for detecting the difference between MI-BP self-monitoring arm and usual care is 90%, with 161 participants per arm. Allowing for 19% attrition based on our previous work in similar patient populations, we plan to recruit 198 participants per arm, for a total of 396 participants. It is anticipated that at the end of trial, both groups will improve on average SBP. We estimate a 10- and 17- point drop in the usual care and MI-BP arms, respectively, at 1 year. Further, a constant between-subject SD of 10 mmHg is assumed, along with an intra-subject correlation of 0.5. With 161 subjects per arm, we can detect a group-by-time interaction with power >95% at 5% level of significance.

### Procedures

**Figure 2:**
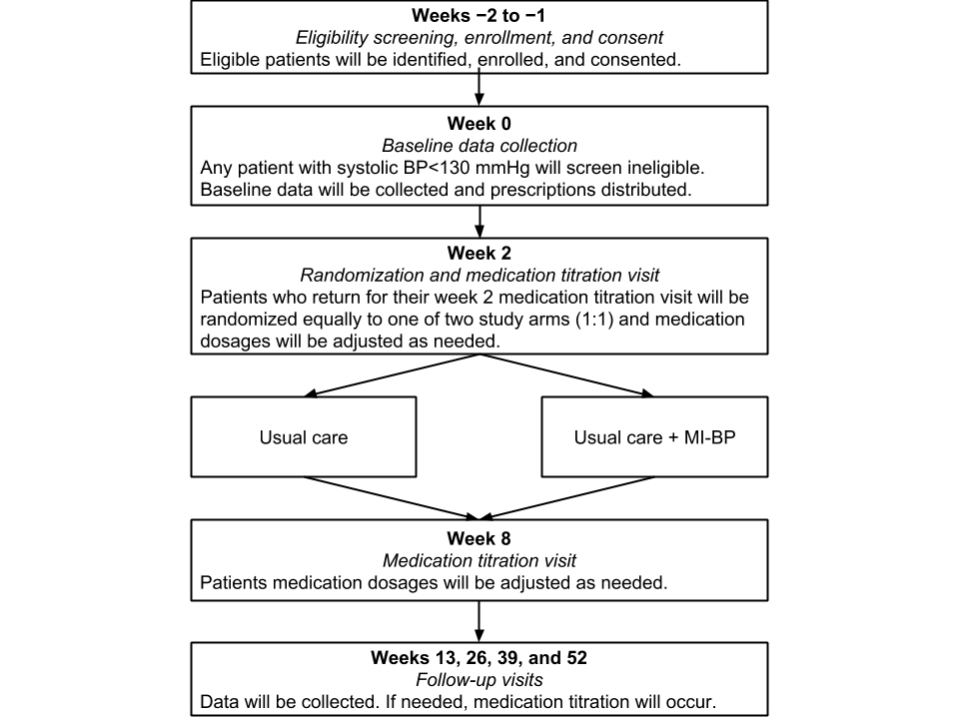
Trial procedure flow diagram.

Once enrolled and consented in the ED (weeks −1 to −2), participants are scheduled for a return visit in 1 to 2 weeks for their baseline appointment (week 0) at the WSU CRSC. As noted above, only those individuals with persistent uncontrolled HTN at this visit are eligible to remain in the study. Baseline data collection also occurs at week 0, and participants are given a prescription for antihypertensive therapy, and referrals to primary care are made by study physicians. For the participants already taking antihypertensive medications and who have an existing relationship with a primary care provider (PCP), we contact their PCP to inform them of our algorithm-based approach to antihypertensive therapy and work to coordinate any medication adjustments. In week 2, participants undergo medication titration, the process of adjusting antihypertensive medication dosages to ensure appropriate and optimal treatment, and are then randomized into 1 of the 2 study arms. Please see Multimedia Appendix 2 for the medication algorithm used in this trial for titration. From our previous unpublished work, we have determined that attrition is greatest between ED recruitment and the first follow-up visit. Thus, by delaying randomization until after this run-in period, we anticipate fewer participants will be lost to follow-up. Moreover, to reduce attrition, at baseline, we collect contact information for up to 3 alternate contacts, who we may call if we are unable to locate the participant to schedule data collection visits. Our staff also heavily rely on telephone calls and text messaging to participant smartphones to communicate as needed to coordinate scheduling, which tend to be the preferred modes of communication among participants. If needed and requested by participants, we also provide transportation to data collection visits. The MI-BP study staff take a participant-centered approach to trial management and are available daily to meet participant needs. Participants are encouraged to call the staff for any questions pertaining to their medications, their BP, technical issues with the intervention, or other issues that arise (see Figure 2 for a flow diagram of trial procedures). Peer-review report from the Center for Scientific Review has been provided in Multimedia Appendix 3.

Randomization is stratified by sex in blocks of equal size, allowing us to investigate sex as an effect-modifying biological variable. To control response fatigue, we created 6 different permutations of the baseline survey, each with a different order of instruments, which are also balanced within blocks. The research assistants responsible for arm allocation are blinded to block size to prevent contamination.

After randomization, all study materials, including any equipment and/or materials, are distributed to the participants according to the treatment arm. Medication titration occurs again at week 8, and at subsequent follow-up visits at weeks 13, 26, 39, and 52 to ensure optimal treatment. Data collection follow-up visits take place at weeks 13, 26, 39, and 52, where a consistent set of study measures is collected—BP, health status, weight, adherence to BP measurements, physical activity, sodium intake, and medication adherence self-monitoring. Technology acceptance measures, including perceived ease of use and perceived usefulness, are also collected.

For medication titration and follow-up visits, patients are instructed to bring their HTN medications with them so pill counts can be conducted. Pill counts are used to avoid prescribing potentially harmful up-titration in antihypertensive therapy for patients with elevated BP at follow-up visits that may be because of medication noncompliance. For patients who self-report not filling prescriptions between visits, or when the expected number of pills found in the participant’s medication bottles (based on dosage and dispense date) suggests a less than 80% medication adherence, we continue with the same regimen without adjustment. We also monitor for any potentially harmful renal or metabolic issues at weeks 0, 26, and 52 and adjust medications accordingly. In addition to the medications, participants are instructed to bring study-dispensed devices so data can be downloaded, and adherence to self-monitoring behaviors can be ascertained.

Finally, to measure sodium intake, at weeks 0, 26, and 52, participants are given supplies to collect 24-hour urine for sodium measurement. This test reveals the amount of sodium ingested in the previous 24 hours and may identify whether sodium intake has been reduced. Study staff collect these specimens directly from the participants at their home to ensure compliance. All medication titration and study follow-up visits are free; however, participants are responsible for the cost of medications, PCP visits, or copays, as applicable.

All the participants are encouraged to contact the study staff for any assistance needed for the study, including assistance for technical and nontechnical matters. This is accomplished through in-app messaging (intervention arm only), as well as through handouts given at randomization and in person at each data collection visit.

### Trial Arm Description

Participants are randomized equally to the 2 treatment arms, which include an enhanced usual care control arm as well as the MI-BP intervention arm.

#### Comparison Group: Enhanced Usual Care

The usual-care participants are given a prescription for antihypertensive medications, printed educational materials on HTN, and a home BP monitor for daily use, but they receive no further intervention (see Table 1); however, the participants take part in all study-specific follow-up visits. Although not all patients with HTN consistently utilize home BP monitoring, it is widely accepted as a guideline-based standard of care, making it appropriate to include in the usual care arm.

**Table 1:** Trial arms.

Components: intervention component	Trial arm	
	Usual care	Usual care + MI-BP
Prescription for hypertension medication	Yes	Yes
Referral to primary care if needed	Yes	Yes
Home blood pressure monitoring	Yes, but no digital tracking tools	Yes, with digital tracking tools via MI-BP app
Physical activity monitoring	No	Yes, with Fitbit or MI-BP app
Sodium intake self-monitoring	No	Yes, with MI-BP app
Medication reminders	No	Yes, with MI-BP app
Patient education materials	Yes, via pamphlet	Yes, with pamphlet or MI-BP app
Goal setting and motivational messages	No	Yes, with MI-BP app
Medication titration and data collection visits at weeks 2, 8, 13, 26, 39, and 52	Yes	Yes

#### Intervention Group: MI-BP, Technology-Enhanced Self-Monitoring

MI-BP participants receive an antihypertensive medication prescription and are asked to use the MI-BP intervention described above for 12 months.

### Measures

Throughout this study, we are collecting a variety of different measures to help determine the feasibility and efficacy of MI-BP including BP, physical activity, sodium intake, and medication adherence. Given the number of measures we are assessing in this trial, our primary data collection visits at baseline and weeks 13, 26, 39, and 52 are lengthy and take about 90 min to complete. To reduce the effect of potential response fatigue because of participant burden, we have divided our surveys into sections focused on measures pertaining to BP, physical activity, and sodium intake, and block orders are randomized among participants. The remaining data collection visits are 30 min or less in duration. While we are collecting a range of measures to ascertain intervention efficacy, our main outcome is differential BP change over time. Other measures will be included as covariates in the modeling of our main outcome and analyzed independently as exploratory end points. In this way, we will be able to capture information on the mediators of our main outcome along with independent effects on specific aspects of self-management.

#### Blood Pressure

We are collecting 2 types of BP data in this trial: in-clinic and home BPs. In-clinic BPs are assessed for every patient, regardless of trial arm, at every study visit by a trained study staff member using a BpTRU BP monitoring device. Home BP data are also collected from the intervention-group participants as a part of their use of the MI-BP app.

#### Physical Activity

A total of 2 different types of physical activity data are collected in this trial. All the participants, regardless of treatment arm, complete the International Physical Activity Questionnaire at baseline and weeks 13, 26, 39, and 52 [27]. In addition, the participants randomized to the MI-BP intervention group are instructed to wear their Fitbit Zip pedometer daily and asked to sync their Fitbit Zip using the Fitbit app at least once per week, which allows the MI-BP app to obtain their physical activity data.

#### Sodium Intake

Sodium intake is measured by 3 different ways. All the participants, regardless of treatment arm, are asked to complete the Block Sodium Screener at weeks 0, 13, 26, 39, and 52 [28], as well as a 24-hour urine sodium test at baseline and weeks 26 and 52. The latter provides an objective measure of the actual total daily sodium intake load. To complete the 24-hour sodium assessment, participants are provided collection materials during study visits and instructed to start their urine collection the following morning. The participants are instructed to start the 24-hour collection time immediately after the first-morning urination, which is discarded and not included in the collection. Participants collect all voided urine for the remainder of the day and night. The next morning, at the same time as day 1, participants collect the first-morning urine and add this to the total, so that a full 24-hour urine has been collected. Participants are instructed to store their specimen in the refrigerator during and after the collection process. Finally, the intervention-group participants are asked to periodically self-monitor their intake of high-sodium foods using a checklist approach, as previously described.

#### Medication Adherence

Medication adherence is measured by 3 different methods to gain a highly comprehensive approach to assess medication adherence, offsetting the weakness of each individual measure in the process.

##### Pill Counts

Participants are asked to bring all HTN medications to follow-up visits at weeks 2, 8, 13, 26, 39, and 52 so that pill counts can be conducted. Participants will be considered adherent if the number of pills remaining in the bottle is within 20% of the expected amount.

##### Self-Reported Adherence

Self-reported medication adherence will be assessed at baseline and weeks 2, 8, 13, 26, 39, and 52, with the Adherence to Refills and Medication Scale, a validated and widely used self-report measure [29].

##### Medication Adherence Assay

**Figure 3:**
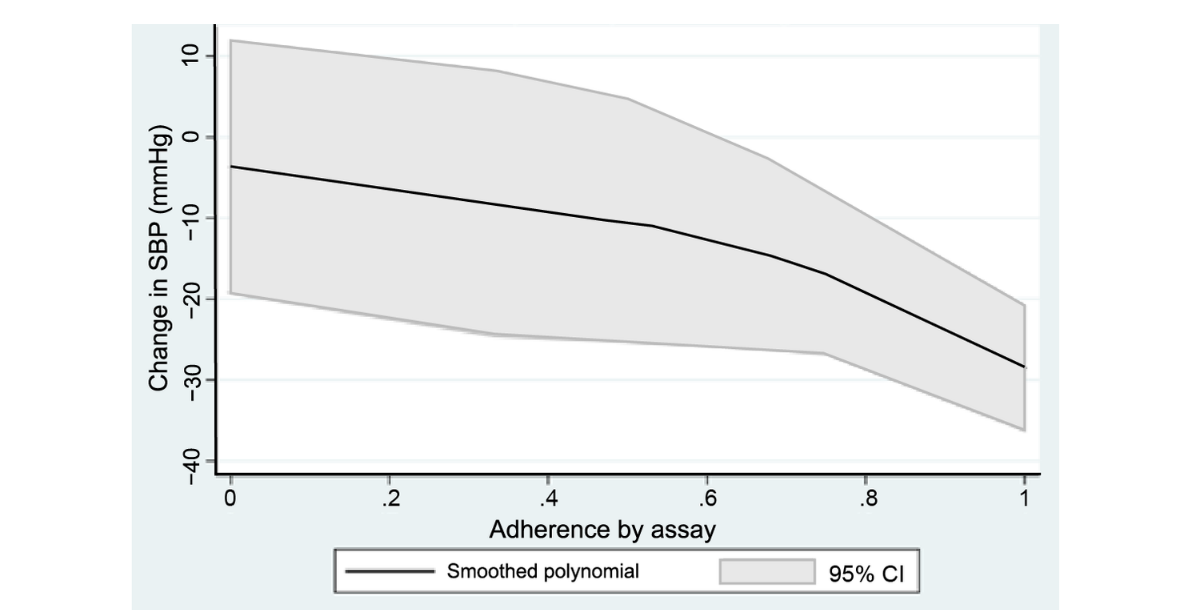
Association between assay adherence and change in systolic blood pressure (unpublished data from previous work). SBP: systolic blood pressure.

Blood samples are drawn at baseline and follow-up visits at weeks 13, 26, 39, and 52, and a liquid chromatography mass spectrometry (LC-MS) assay developed by Precera Bioscience, Inc (Brentwood, TN) is used to detect several hundred prescription medications, over-the-counter medications, and medication metabolites in each patient sample received (including all drugs incorporated into our treatment algorithm) in participant blood samples. This serves as a direct, objective assessment of medication presence and levels to measure medication adherence. This assessment will be compared with other measures of medication adherence. The assay imparts limited burden on participants, as it requires only 100 microliters of serum or plasma. Unpublished data from our previous work have shown that there is a negative relationship between assay adherence and change in SBP among patients prescribed less than or equal to 3 antihypertensive medications (Figure 3) [30].

#### Self-Efficacies for Behavior Change

We collect various measures of self-efficacy for our target behavior changes at baseline and weeks 13, 26, 39, and 52, including physical activity via the Exercise Self-Efficacy Scale [31], medication adherence via the Medication Adherence Self-Efficacy Scale [32], and diet using an investigator-developed 11-item instrument assessing confidence in reducing sodium consumption, avoiding high-fat foods, avoiding sugar-sweetened beverages, and improving vegetable and legume intake.

#### Additional Health Measures

In addition to measures focused on specific behavior changes, we will also measure patient activation with the Patient Activation Measure [33], HTN knowledge with the 14-item Hypertension Evaluation of Lifestyle and Management Knowledge Scale [34], and functional status using the 12-Item Short-Form Health Survey (SF-12) at baseline and weeks 13, 26, 39, and 52 [35]. Additionally, we will measure health literacy via the 7-item Rapid Estimate of Adult Literacy in Medicine-Short Form at baseline [36], a modified Sugar-Sweetened Beverages measure at baseline and weeks 26 and 52 [37], and patient perceptions of the LC-MS assay for medication adherence, using investigator-developed questions, at week 52.

#### Technology-Related Measures (MI-BP Group Only)

To help put into context the intervention-group outcomes, we will assess several different technology-related measures, including technology acceptance (among the MI-BP participants only) via measures adapted from the Technology Acceptance Model by Davis that looks at participant perceptions of the perceived ease of use and perceived usefulness of MI-BP at weeks 13, 26, 39, and 52 [38]. We will also assess participant perceptions of the MI-BP intervention. Finally, MI-BP utilization will be assessed through usage log analysis at the end of the study.

### Statistical Analysis Plan

#### Analysis of Blood Pressure Control

To determine the effect of MI-BP on BP at 1 year (aim 1) and to determine whether BP control is better in intervention-group participants compared with controls (hypotheses 1a), we will conduct a comparative analysis using a logistic regression with BP control as the outcome and study arm as the primary factor, controlling for demographic characteristics such as age, gender, medical history, baseline levels of functional status, health literacy, HTN knowledge, self-efficacy, and baseline BP at randomization. To determine whether mean SBP is better in the intervention group, compared with controls (hypothesis 1b), differential reduction in the coprimary outcome of SBP will be assessed in a linear mixed-effects regression framework, with time (baseline and end-of-trial), group, and time-by-group interaction as the primary independent variables. The subject-level characteristics used in the analysis of the primary outcome will be controlled in the regression model.

To determine the effect of MI-BP on secondary outcomes (aim 2), we will also use linear mixed-effects regression models with the secondary outcomes of interest as the dependent variables and week (as a continuous variable), study group, and week-by-group interaction as the primary independent variables. All appropriate model diagnostics will be carried out.

During data analysis, we will statistically address missing data resulting from incomplete data collection or participant attrition using the multiple imputation technique. Missing values will be imputed by repeatedly and iteratively fitting a sequence of regression models; it is a technique that is flexible in allowing different types of variables (categorical and continuous) to be imputed together without requiring any multivariate joint distributional assumption. Missing values are sequentially updated using bootstrap or Markov Chain Monte Carlo based on multiple regression models with other variables as covariates. This procedure will be conducted for 10 repetitions or cycles, a number considered adequate for most applications, thereby constructing an *imputed* dataset. Results from the 10 regressions will be combined with the imputed data using Rubin’s formula [39].

#### Analysis of Cost-Effectiveness Data

Cost-effectiveness studies focused on mHealth interventions, specifically within the context of HTN, are lacking from the literature [40]. We will evaluate the cost-effectiveness of MI-BP using data from within the trial (aim 3); however, since HTN is a long-term chronic disease, the observed outcomes for the patients may not capture the entire benefit of the intervention. As such, we will also use a modeling approach to simulate patient lifetime outcomes, given their health status and trajectories at the end of the trial. We hypothesize that MI-BP will be cost-effective compared with usual care.

Costs will be prospectively collected from DMC, which serves most of the health needs of enrolled individuals. For the within-trial cost-effectiveness analysis, we will use ED, pharmacy, outpatient, estimated patient costs, and hospital cost data. Quarterly SF-12 assessments will be converted into utility measures using the methods of Brazier et al [41] and aggregated over the year to calculate quality adjusted life years (QALYs), a standard health-economic measure to compare across interventions. Total costs and total QALYs will be calculated for both arms. The MI-BP arm will be compared with usual care using an Incremental Cost-Effectiveness Ratio, a measure that compares health value for money. Bootstrapping will be used to assess uncertainty around the mean estimates, and it will be used to create cost-effectiveness acceptability curves [42].

For the long-term cost-effectiveness analysis, we will create a mathematical model of long-term quality of life and mortality because of HTN, which will be based upon other models in the literature, such as the approach used by Smith-Spangler et al [43], which modeled the relationship between SBP and long-term heart attack and stroke using a logistic risk function based on the Framingham data. Costs of disease states such as heart attacks and strokes will be taken from extant literature and databases such as the Medical Expenditure Panel Survey and Healthcare Cost and Utilization Project of the Agency for Healthcare Research and Quality. Health utilities will be taken both from the utilities collected and the medical literature (particularly for heart attacks and strokes). The models will begin with the health state of the participants at the end of the trial and will model health outcomes into the future. We will explore various assumptions about the durability of BP changes (eg, continuing improvement, stable BP, regression to prestudy BP levels). These cost-effectiveness analyses will follow standard guidelines for a *reference case* analysis, while the modeling approach and analysis will follow recent guidelines [44-46].

In addition to analyses for our stated aims, we also intend to conduct analyses to look at the patterns of MI-BP use that are associated with improved outcomes. For each of our self-monitoring behaviors (BP, physical activity, sodium intake, and medication adherence), we will calculate an overall utilization rate by dividing the number of logged data points by the total number of expected data points. Adherence to behavioral self-monitoring uptake for each activity will be compared within the MI-BP arm by means of count regression with the number of adherence instances (among the repeated observations) as outcome. Models will be controlled for variables similar to those in aim 1. To investigate whether better adherence to the self-monitoring uptake corresponds to an improvement in BP, we shall use a mixed-model analysis similar to that in aim 1 with BP measure as outcome and adherence as time-dependent binary covariates. Different self-monitoring behavioral components will simultaneously be used in the same model.

## Results

The recruitment period for the study began in January 2018 and was met with challenges because of overly restrictive inclusion criteria. Please see our section on Limitations for further discussion. After amending our protocol, recruitment efforts have become more fruitful, and this study is expected to conclude in 2021.

## Discussion

### Principal Findings

At the conclusion of this study, we expect to be able to demonstrate the efficacy of using a novel and innovative multicomponent mHealth approach for supporting HTN management in a community that has exceedingly high prevalence of untreated chronic diseases. By incorporating mobile devices in a population that has high smartphone adoption rates, mHealth interventions may be superior compared with usual care methods. We anticipate that by focusing on multiple health behaviors, such as diet and exercise, along with promoting self-monitoring, we will reduce HTN-related disparities in African Americans with uncontrolled BP. We will be able to document the efficacy of MI-BP and learn from our participants how to overcome barriers to BP control, ultimately reducing deaths from HTN-related cardiovascular disease.

### Comparison With Previous Work

Although mHealth approaches for targeting HTN have been previously reported, most are focused on single behavior interventions that utilize simple platforms such as text messaging [40]. Moreover, despite the promise of mHealth for HTN, a recent scientific statement of the AHA on the use of mHealth for cardiovascular disease prevention found only 13 RCTs of sufficient quality focused on mHealth to promote BP control, of which only 4 utilized smartphones for intervention delivery. Furthermore, of the 69 total studies identified for cardiovascular disease prevention, it was noted that most relied on short message service text message and internet-mediated delivery modalities, and few used more advanced mHealth approaches, such as those incorporated into MI-BP. Although the AHA scientific statement acknowledged promise for mHealth approaches to reduce SBP, several limitations of the available research were noted, including a lack of understanding about which intervention components led to behavior changes, a lack of understanding of factors that contribute to technology use, and short-term (less than 6 months) follow-up [47].

This study builds upon our previous work with BPMED, a single-behavior change intervention, delivered via text messaging, to target medication adherence in this same population [15]. Our work with BPMED demonstrated the feasibility and acceptability of using a mobile approach within this target population. This MI-BP trial extends our previous work by using a more robust platform that targets many of the recommended self-care behaviors for managing HTN, and it is in line with the more sophisticated mHealth interventions for HTN that are missing from the literature [40]. There has been some acknowledgement in the literature that more sophisticated apps, with more comprehensive feature sets, may be more effective in lowering BP [48]. Furthermore, although this MI-BP trial is just 1 of several ongoing trials targeting HTN with mobile approaches, to the best of our knowledge, this is the only study that seeks to understand how such an intervention may affect African Americans in urban environments, where HTN-related health disparities are common.

### Limitations

Perhaps the largest limitation of this study is the risk of loss of participants after initial enrollment. To address this, a staged screening method is used to identify truly interested participants, and we will oversample by 19%. Moreover, we use a distributed incentive system, rewarding study visit completion. Additional retention strategies include obtaining contact information for the participant and up to 3 friends or relatives who can help locate the participant. The other main limitation relates to our plan for relatively aggressive participant recruitment. Quickly after launching recruitment, and before the publication of this protocol, our study team saw that we were not accruing participants at the rate that we had hoped for. After reviewing our screen-fail logs, we identified that our biggest barriers for enrollment were BP criteria and age. As such, with the support of our Data Safety Monitoring Board we submitted protocol amendments with our IRBs, as well as with Clinicaltrials.gov, to relax eligibility age criteria (from 25-55 years to 25-70 years) and SBP criteria (from >160 mmHg to ≥135 mmHg at screening, and from >140 mmHg to >130 mmHg at baseline) and to add a recruitment site. In addition to helping spur recruitment, these changes were also made to better reflect the new AHA BP guidelines for detecting and managing HTN [1]. Finally, generalizability will be limited by virtue of our target population; however, our primary goal is to improve BP control and limit the consequences of HTN on a disproportionate risk population.

### Conclusions

As the first of its kind, MI-BP was designed to test the efficacy and acceptability of a multicomponent mHealth approach to help African Americans with uncontrolled BP modify their lifestyle to better manage their HTN. We expect to lay the foundation to sustainably reduce HTN-related health disparities through better integration of multiple behavior self-monitoring. If the MI-BP trial is effective at reducing BP in our target population, it would provide solid evidence to support the development of similar mHealth-based interventions aimed at improving HTN control among vulnerable patients. While our cohort is exclusively African American and replication of findings would be needed in a broader patient population before widespread implementation, data from MI-BP would be among the first to be obtained from the ED, supporting the viability of this often-overlooked setting as a delivery point for chronic disease management. Finally, the success of MI-BP would support future efforts to create health policies that seek expanded coverage and reimbursement for mHealth initiatives focused on bolstering self-monitoring for HTN and other chronic conditions.

## Appendix

Multimedia Appendix 1 Sample messages.

Multimedia Appendix 2 Titration algorithm.

Multimedia Appendix 3 Peer-reviewer report from the Center for Scientific Review.

## Abbreviations

AHA: American Heart Association
BP: blood pressure
CRSC: Clinical Research Service Center
DMC: Detroit Medical Center
DRH: Detroit Receiving Hospital
ED: emergency department
HTN: hypertension
IRB: institutional review board
LC-MS: liquid chromatography mass spectrometry
mHealth: mobile health
PCP: primary care provider
QALY: quality adjusted life year
RCT: randomized controlled trial
SBP: systolic blood pressure
SF-12: 12-Item Short-Form Health Survey
SGH: Sinai-Grace Hospital
WSU: Wayne State University
