# Supplementary material for: Improving Blood Pressure Among African Americans With Hypertension Using a Mobile Health Approach (the MI-BP App): Protocol for a Randomized Controlled Trial
Source: JMIR Res Protoc. 2019 Jan 25;8(1):e12601. doi: 10.2196/12601 (PMC6367671; doi:10.2196/12601)
Supplement: Multimedia Appendix 1 [file resprot_v8i1e12601_app1.pdf]

Appendix A: Sample messages from the MI-BP app by type

Message Types	Example 1	Example 2
**Educational**		
Physical activity	To help make exercise a regular habit, set clear and realistic short-term goals. Don't try to do it all overnight!	If you are trying to make exercise a habit, try keeping yourself on a regular schedule so that the habit sticks. Doing something over and over is key to building a habit.
Nutrition	When eating out, try choosing fruit and vegetables as side dishes instead of chips or fries, which contain high amounts of sodium. Reducing your sodium intake can help lower your blood pressure	When using canned foods, such as beans or tuna, rinse them with water for one minute to remove extra sodium.
Medication Adherence	If you're having trouble remembering to take your blood pressure medicine, write yourself a note. Post it somewhere you look often, like the bathroom mirror.	Make sure to pack enough blood pressure pills when you're away from home. Your high blood pressure does not take a vacation!
BP Monitoring	Measuring your blood pressure at the same time every day is the best way to see your blood pressure numbers improve over time.	Remember to sit still with your feet flat on the floor for at least 5 minutes before taking your blood pressure. This will help you get a proper reading.
Hypertension	High blood pressure can damage blood vessels in your eyes and lead to vision problems, including blindness. Keep up your healthy habits to prevent damage.	You can have high blood pressure even if you don't feel sick. Make sure you take your medicine, eat well, and get regular exercise to help lower your blood pressure.
Devices	Help make exercise a regular habit by tracking your progress. MI-BP will really help with this! Be sure to sync your Fitbit often.	Remember that your blood pressure monitor is a great tool to helping you achieve your goals. Use it often to monitor changes in your blood pressure over time.
Other	Did you know that reducing the amount of caffeine you drink each day can help lower your blood pressure?	Drinking alcohol can increase your blood pressure. Try to limit yourself to 1 drink per day for women, or 2 drinks per day for men to help keep your blood pressure under control.
**Motivational**		
	Make choices that are right for you, like taking your medicine, eating well, and exercising. Choosing to put your health first benefits you and your loved ones.	Lowering your blood pressure even a little can help improve your long-term heart health. Every bit counts!
**Tailored**		
Lack of time	Is it hard to find time to exercise? Plan ahead! Before your week starts, make appointments with yourself to be active and stay on track.	Short on time? Exercise doesn't have to take hours out of your day. A 10-30-minute walk is a good way to be active when your day is full.
Social influence	Turning exercise into a social activity is a good way for your friends and family to support you in your goal of being more active. Plus, everyone benefits!	Get outside and play with your kids or other young people in your life. Kids love to have adults to run around with, and you'll be the favorite adult in the room!
Lack of energy	Believe it or not, being active can help you have more energy… even if you are already tired! Feeling sluggish? Do something active for 10 minutes and then see how you feel.	Make sure you are getting enough sleep at night. It is hard to want to work out when you are tired, so do yourself a favor and ensure you are well rested.
Lack of willpower / motivation	After exercising for a few days, pay attention to how you feel mentally. Focusing on how exercise can help boost your mood and general outlook on life can be a motivator to keep with it.	Don't focus on the aspects of working out that you don't like. Instead, focus on the positive aspects to keep you motivated to succeed.
Fear of injury / pain	Know your body. For example, if you have chronic knee pain, avoid exercises that strain your knees such as running on a treadmill, stair stepper machines, or squats.	To lower your chance of injury, start your workouts slow and slowly increase your intensity and duration; particularly if you haven't been very active in a while.
Lack of resources	You don't need expensive equipment to exercise. Household items like canned vegetables or jugs filled with water, as well as bungee cords are often good substitutes for weights and resistance bands.	YouTube videos are an easy and free way to try out new exercise programs that can be done in the comfort of your own home.

Family obligations	Instead of a movie or game night with the family, try planning a family activity that keeps everyone active such as basketball, a bike ride, or even a family walk.	Caring for others can put a strain on your own health and well-being. Exercise is a great way to relieve stress and can give you the energy and mental resolve to better care for others.
Weather conditions	There are lots of indoor places that can keep you active when the weather is bad. Try walking in a large store, signing up for an indoor sports league, or taking classes at a gym or community center.	Don't let the weather keep you from exercising. Be prepared by having a set of exercise routines you can do at home. A little planning ahead can keep you active!
Depression	If you are feeling depressed, pick a small attainable exercise goal. If all you think you can do is go for a short walk, start there and see if you can add more tomorrow.	Finding workouts you enjoy can help you stick with your exercise routine, even when you aren't feeling up to it. If you plan to do things you know you hate, you aren't likely to stick to the plan!
Accountability / external motivation	Making plans to work out with other people, either in a small group or in a class, is a great way to keep you accountable and sticking to your workout routine.	Writing down your exercise goals can help keep you accountable to yourself. If you write down your plans, you are more likely to stick to them.
Disability	If you have limitations on the types of physical activities you can do, find something that you can do that you enjoy. It doesn't matter what you do to stay active, what matters is that you do it!	Exercising doesn't mean just working out at high intensity for a long time. Small chunks can give the same benefits. Try 3 chunks of 10 minutes throughout your day instead of 30 minutes.
Sync reminder for Fitbit	We haven't received any data from your Fitbit in the last week. Be sure to wear your Fitbit and open your Fitbit app every day.	We haven't received any data from your Fitbit in a while. Be sure to wear your Fitbit and open your Fitbit app every day. If you are having problems with your Fitbit, please call us at XXX-XXX-XXXX.
Sync reminder for BP cuff	We haven't received any blood pressure data from you in the last week. Please be sure your MI-BP app is open when you use the cuff. You can also use the + icon to add your reading directly	We haven't received any blood pressure data from you in a while. If you are having problems with your cuff, please call us at XXX-XXX-XXXX.
Sodium logging results	It looks like you didn't make your goal for the daily number of high sodium foods this logging period. Let's try again in two weeks. Click here if you need help finding good food substitutes.	Good work on meeting your sodium goal!
Sodium goal	On average, you have been eating XX high sodium foods per day. Your goal for the next period is XX high sodium foods per day. If you need help finding good substitutions, click here.	N/A
Step count goal	Your first step count goal is X,XXX steps per day. Please tap to see your data. You will receive a new goal on Sunday based on the data you sync this week. Please remove the sticker from your Fitbit	Your new step count goal is X,XXX steps per day for the week. Please tap to see your data. You will receive a new goal next week based on the data you sync this week
**Medication Reminder**		
Reminder	Just checking in - did you remember to take your blood pressure medicine today?	Be sure to take your blood pressure medicine at your usual time every day to protect your long-term health.
