# Supplementary figures and images for: Improving Blood Pressure Among African Americans With Hypertension Using a Mobile Health Approach (the MI-BP App): Protocol for a Randomized Controlled Trial
Source: JMIR Res Protoc. 2019 Jan 25;8(1):e12601. doi: 10.2196/12601 (PMC6367671; doi:10.2196/12601)

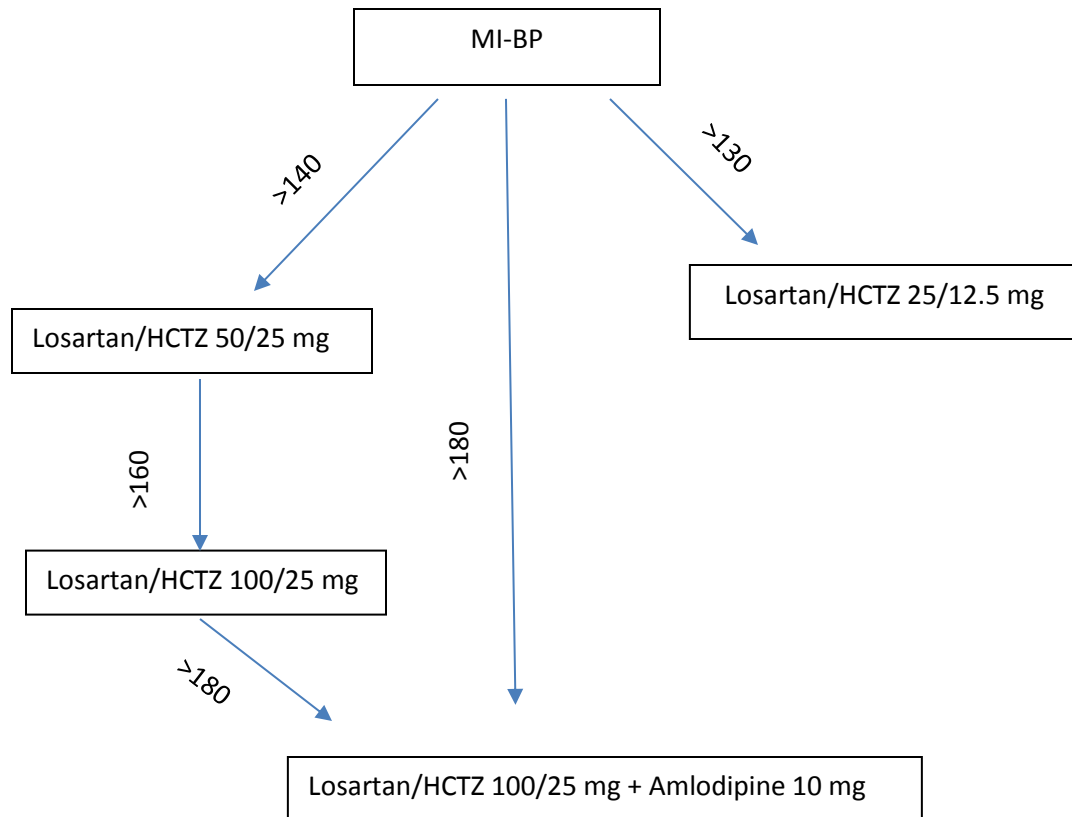

Supplement: Multimedia Appendix 2 [file resprot_v8i1e12601_app2.pdf]
