# Supplementary material for: Improving Blood Pressure Among African Americans With Hypertension Using a Mobile Health Approach (the MI-BP App): Protocol for a Randomized Controlled Trial
Source: JMIR Res Protoc. 2019 Jan 25;8(1):e12601. doi: 10.2196/12601 (PMC6367671; doi:10.2196/12601)
Supplement: Multimedia Appendix 3 [file resprot_v8i1e12601_app3.pdf]

SUMMARY STATEMENT
(Privileged Communication)

PROGRAM CONTACT:
Paula Einhorn
(301) 435-0563
einhornp@nhlbi.nih.gov

Release Date: **10/27/2015**

Application Number: **1 R01 HL127215-01A1**

Principal Investigator

BUIS, LORRAINE R PHD

Applicant Organization: UNIVERSITY OF MICHIGAN

Review Group: **BMIO**
Behavioral Medicine, Interventions and Outcomes Study Section

Meeting Date: **10/05/2015**
Council: **JAN 2016**
Requested Start: **04/01/2016**
RFA/PA: **PA13-292**
PCC: **HHCP N**

Project Title: **MI-BP: mHealth to Improve Blood Pressure Control in Hypertensive African Americans**
SRG Action: **Impact Score: 40 Percentile: 24**
Next Steps: **Visit http://grants.nih.gov/grants/next_steps.htm**
Human Subjects: 30-Human subjects involved - Certified, no SRG concerns
Animal Subjects: 10-No live vertebrate animals involved for competing appl.
Gender: **1A-Both genders, scientifically acceptable**
Minority: **2A-Only minorities, scientifically acceptable**
Children: **3A-No children included, scientifically acceptable**
Clinical Research - not NIH-defined Phase III Trial

Project Year	Direct Costs Requested	Estimated Total Cost
1	482,765	750,890
2	497,492	773,797
3	497,787	774,256
4	477,340	742,452
TOTAL	1,955,384	3,041,395

ADMINISTRATIVE BUDGET NOTE: The budget shown is the requested budget and has not been adjusted to reflect any recommendations made by reviewers. If an award is planned, the costs will be calculated by Institute grants management staff based on the recommendations outlined below in the COMMITTEE BUDGET RECOMMENDATIONS section.

EARLY STAGE INVESTIGATOR, NEW INVESTIGATOR

1R01HL127215-01A1 Buis, Lorraine

NEW INVESTIGATOR
EARLY STAGE INVESTIGATOR

RESUME AND SUMMARY OF DISCUSSION: This application evaluates the efficacy of a comprehensive, multiple health behavior change mobile intervention that promotes daily intensive self-monitoring of blood pressure, physical activity, sodium intake, and medication adherence among African Americans with uncontrolled HTN. The investigative team and environments are both strong. It is innovative as it will test a mHealth tool that allows for self-monitoring of multiple health behaviors and targets an at risk population. However, during discussion a number of weaknesses were noted: details regarding the tailored in-app messages are not provided; rationale for the 3-arm design is weak; lack of pilot data supporting the feasibility of self-monitoring to promote improved BP control; an underdeveloped intervention protocol, and it was also thought that the applicants were only partially responsive to prior critiques. In summary, the committee concluded that the potential overall impact was moderate.

DESCRIPTION (provided by applicant): Hypertension (HTN), one of the most important cardiovascular risk factors, affects more than 78 million Americans. Compared to other races/ethnicities, African Americans are more likely to develop HTN and have lower rates of blood pressure (BP) control, increasing risk of premature cardiovascular morbidity and mortality. African Americans are also more likely to utilize the emergency department (ED) for ambulatory care, a factor strongly linked with adverse cardiovascular events among patients with HTN. This high ED utilization may reflect poor access to primary care, and suggests the challenge people face regarding self-management. Because BP is routinely measured in the ED, it is an ideal setting to both identify patients with uncontrolled HTN and intercede, particularly in African American communities where regular interaction with the health care system may be lacking. Recommendations to improve HTN-related outcomes have been consistent for decades: maintain a healthy weight, reduce daily sodium intake, increase physical activity, and comply with antihypertensive therapy as prescribed. Despite tremendous evidence supporting these recommendations, facilitating the necessary behavior changes in patients with HTN remains a challenge, especially in African Americans who reside in urban, under-resourced settings. Daily intensive self-monitoring is efficacious and is often the cornerstone of many behavior change interventions; however, long-term sustainment can be difficult to achieve, especially in minority populations residing in urban, under-resourced communities. Mobile health (mHealth) has demonstrated success with behavior change, and may increase long-term self- monitoring. Given high cell phone adoption rates in minority communities, and higher rates of reliance on mobile Internet access, mHealth strategies are particularly well suited to urban African American populations. Currently, there exists traction among physicians and patients for mHealth HTN interventions, but such an approach to improve BP in hypertensive patients has not been tested. This study seeks to improve HTN-related outcomes in a cohort of African Americans with uncontrolled HTN between the ages of 25 and 55, and is guided by three specific aims. Aim 1: We will determine the efficacy of MI-BP, a comprehensive, multiple health behavior change mobile intervention, on BP control at one year in a randomized controlled trial (RCT) of the intervention compared to paper-based self- monitoring and usual care controls. Aim 2: We will determine the effect of MI-BP on secondary outcomes (physical activity, sodium intake, medication adherence) compared to paper-based self- monitoring and usual care controls, in a one year RCT. Aim 3: We will evaluate the cost-effectiveness of MI-BP compared to paper-based self-monitoring and usual care controls.

PUBLIC HEALTH RELEVANCE: Hypertension (HTN), one of the most common and important cardiovascular risk factors, disproportionally affects African Americans, as they experience lower rates

of blood pressure control, a younger age at onset, and more HTN-related complications, increasing risk of premature cardiovascular morbidity and mortality. This project tests the efficacy of MI-BP, a comprehensive, multiple health behavior change mobile intervention that promotes daily intensive self-monitoring of blood pressure, physical activity, sodium intake, and medication adherence among African Americans with uncontrolled HTN, with the goal of improving blood pressure and self-care adherence measures.

CRITIQUE 1

Significance: 2
Investigator(s): 3
Innovation: 4
Approach: 4
Environment: 2

Overall Impact: This is the first re-submission of an application that addresses the significant problem of uncontrolled hypertension in a disparities population, African Americans, who are disconnected from primary care, with an mhealth intervention to enhance self-monitoring. If successful, components of the mhealth-based intervention under study have the potential to be an effective and scalable way to enhance blood pressure control. The PI is an ESI with an impressive publication record and some previous funding in the mhealth area. The proposed intervention is moderately innovative, but will be applied to a novel understudied population. The revised application addresses some of the major weaknesses noted on the first submission. This revision clarifies the preliminary data, which demonstrates the feasibility and acceptability of the intervention, as well as the team’s ability to recruit and retain the target population. Enthusiasm for this improved application would be higher if more detail about the content of the health messages was provided, as mentioned in the prior critique. Because this complex intervention seems to hinge on the tailored health messages and prompts, examples of health messages would be helpful to assess this important component of the intervention. The research team is well equipped to carry out the proposed work in the ideal scientific environment provided by the complementary research team, and the clinical and institutional resources.

1. Significance:

Strengths

- The application addresses a significant problem that is an important, remediable source of health disparities.
- Components of the mhealth-based intervention under study have the potential to be an effective and scalable way to enhance blood pressure control.
- The population under study, African American patients, who preferentially use the ED, and as well as smart-phone technology appears to be group that can gain substantial benefit from the proposed intervention.

Weaknesses

- The weaknesses regarding the participant burden imposed by the intervention have been addressed; the intervention seems to be scalable, and thus potentially significant.

2. Investigator(s):

Strengths

- The PI is an ESI with an impressive publication record and some previous funding in the mhealth arena.
- The supporting team is well suited to support this ESI and ensure successful implementation of the trial. Dr. Levy is well known in ER-based hypertension research, and has a considerable investment in this project.

Weaknesses

- Given the target population will have poorly controlled stage 2 hypertension and possibly resistant hypertension, consideration should be given to including a clinical hypertension specialist on the team.

3. Innovation:

Strengths

- While IMB-based mhealth interventions are not new, the application to this specific population of high ED-utilizers is novel.
- Serum monitoring of medication-levels is a novel.
- The addition of the fitbit platform to the foundation of text messaging of health messages is innovative.

Weaknesses

- It is difficult to ascertain the innovation of the health messages, as still, no examples were provided.

4. Approach:

Strengths

- Randomized design with a 2-week run-in period to account for regression to the mean is strength.
- Scheduled titration visits to ensure aggressive bp management is a strength.
- Preliminary data demonstrates feasibility and ability of team to recruit and retain the target population.
- Multiple validated ways of assessing adherence to behaviors is strength.
- Detailed self-monitoring protocols for target behaviors are a strength.

Weaknesses

- No discussion of the acceptability of serum drug testing as a measure of medication adherence in this population.
- The self-monitoring and desired behavior changes seem to hinge greatly on the prompts and health messages, which are not well described.

5. Environment:

Strengths

- Strong research environment at U of M, and Dr. Levy's clinical research center.

Weaknesses

- None noted.

Protections for Human Subjects:

Acceptable Risks and/or Adequate Protections

Data and Safety Monitoring Plan (Applicable for Clinical Trials Only):

Acceptable

Inclusion of Women, Minorities and Children:

- Sex/Gender: Distribution justified scientifically
- Race/Ethnicity: Distribution justified scientifically
- Inclusion/Exclusion of Children under 21: Excluding ages < 21 justified scientifically
- All African American, 56% female, 44% male

Vertebrate Animals:

Not Applicable (No Vertebrate Animals)

Biohazards:

Acceptable

Resubmission:

- Partially responsive to the major critiques

Budget and Period of Support:

Recommend as Requested

CRITIQUE 2

Significance: 4
Investigator(s): 3
Innovation: 5
Approach: 7
Environment: 1

Overall Impact: This resubmission application from an NI/ESI proposes a three-arm RCT to test the efficacy of a mobile health intervention (MI-BP) to facilitate self-monitoring of multiple behaviors (home BP monitoring, physical activity, sodium intake, and medication adherence) with the goal of improving blood pressure management, patient activation, and self-care among African Americans with uncontrolled hypertension. Recruitment in the ED is a strength. The potential to address a major public health problem such as uncontrolled HTN in African Americans who are more likely to be disconnected from primary care and instead use the ED as a routine source of care is attractive. However, the investigators failed to adequately address many of the major weaknesses raised in prior reviews, including, but not limited to, a weak rationale for the 3-arm design, a lack of direct pilot data supporting

the feasibility and potential effectiveness of self-monitoring of multiple health behaviors to promote improved BP control, an underdeveloped intervention protocol, and questionable measurement methods of several outcomes. These persistent weaknesses significantly diminish the potential overall impact of the study which is judged to be low to medium.

1. Significance:

Strengths

- Uncontrolled HTN is a major public health problem with blacks disproportionately affected, which contributes downstream disparities in CVD morbidity and mortality.
- Blacks are more likely to be disconnected from primary care and instead use the ED as a routine source of care.
- Addressing these issues through a mobile technology intervention has potential for significant benefits.

Weaknesses

- The investigators did not adequately address the previous concern relating to the sustainability and implementation potential of the intervention to promote self-monitoring of multiple health behaviors in African Americans seen in the ED.
- A critical review of the mHealth intervention for BP management and control is missing.

2. Investigator(s):

Strengths

- The PI is an NI/ESI with a good publication and funding record in relevant areas.
- Her collaborators bring needed complementary expertise.

Weaknesses

- The investigators switched to the Vibrent mobile intervention platform but did not provide a rationale or any evidence of prior collaboration with the company.

3. Innovation:

Strengths

- Using a mHealth intervention to engage African American patients who rely on ED for BP care is innovative.
- Serum monitoring of medication adherence is innovative.

Weaknesses

- Significant details of the intervention remain unspecified or underdeveloped, which was a major concern noted in prior reviews. As described, the mobile intervention is nothing more than using an app (Vibrent) for self-monitoring and text messaging. This is not novel. The AHA scientific statement on mHealth interventions published in Circulation in 8/2015 found studies with very similar features for the prevention and control of CVD risk factors, including hypertension. Two consistent shortcomings of these studies are the lack of theoretical underpinnings for technology use and the lack of maintenance of intervention effects, if any observed. The same concerns apply to this study.

4. Approach:

Strengths

- Recruitment of African Americans with uncontrolled HTN in the ED and evidence of the team's ability to do so in prior studies.
- Inclusion of cost-effectiveness analysis as a specific aim.

Weaknesses

- Even though the proposed study was labeled as a "pragmatic trial," it wasn't designed as such. Numerous exclusion criteria, a run-in period to exclude noncompliant patients before randomization, and team physicians serving as treating physicians are just a few of many design elements that make this more of an explanatory/efficacy trial than a pragmatic trial. The apparent lack of a clear understanding of the explanatory-to-pragmatic continuum in study designs is concerning.
- A 3-arm randomization design remains poorly justified. The investigators stated that the paper-based self-monitoring arm is needed for accomplishing the cost-effectiveness aim, but it is left unexplained why this would be the case. Prior studies, such as that by Burke et al. AJPM 2012, have demonstrated that the modality of self-monitoring (mHealth vs. paper) is less important than the behavior of self-monitoring itself. More research that is needed should be on how best to tailor the modality to individuals and what strategies can promote long-term maintenance.
- The proposal offers no theoretical framework for technology use and adoption.
- Critical details of the mHealth intervention are missing. For example, it is unclear how goal setting and motivational messages will be implemented and what is involved. The shift to Vibrant app is not justified and the features of this app are not adequately described.
- Pedometers are used as intervention tools in the two intervention groups. It is flawed to propose the use of these as the physical activity outcome data anyway, let alone the usual care group will not have pedometer data. Assessing physical activity by the BRFSS questionnaire is inadequate.
- 24-hour urine testing for sodium levels is proposed but no protocol provided. This is a type of test prone to measurement errors unless a standard protocol is strictly followed.
- The effects assumed in the sample size calculations are not substantiated, and the use of a one-sided test is ill advised.

5. Environment:

Strengths

- Excellent environments at the collaborating institutions for the work proposed.

Weaknesses

- None noted.

Protections for Human Subjects:

Acceptable Risks and/or Adequate Protections

Data and Safety Monitoring Plan (Applicable for Clinical Trials Only):

Acceptable

Inclusion of Women, Minorities and Children:

- Sex/Gender: Distribution justified scientifically
- Race/Ethnicity: Distribution justified scientifically
- Inclusion/Exclusion of Children under 21: Excluding ages < 21 justified scientifically
- 56% women; all African Americans

Vertebrate Animals:

Not Applicable (No Vertebrate Animals)

Biohazards:

Not Applicable (No Biohazards)

Resubmission:

- This resubmission application was submitted by a promising NI/ESI. Unfortunately, many of the noted weaknesses in prior reviews were not addressed at all or only incompletely.

Budget and Period of Support:

Recommend as Requested

CRITIQUE 3

Significance: 2
Investigator(s): 1
Innovation: 2
Approach: 4
Environment: 1

Overall Impact: This project tests the efficacy of a comprehensive, multiple health behavior change mobile intervention that promotes daily intensive self-monitoring of blood pressure, physical activity, sodium intake, and medication adherence among African Americans with uncontrolled HTN. The investigative team and scientific/clinical environments are both strong. The study is considered innovative as it will test a mHealth tool that allows for self-monitoring of multiple health behaviors, targets an at risk population, and recruits that population with a smart approach. There are a few weaknesses in the approach but most are considered minor. The group was very responsive to the previous round of revisions. Overall, the impact for this project is considered high.

1. Significance:

Strengths

- This study addresses an important issue of promoting self-management behaviors for hypertension control amongst a population at risk for poorer self-management behaviors.
- The study takes a unique approach of focusing on multiple health behaviors.

- Should the aims be met, the study would provide clinicians and practitioners an effective intervention approach designed to improved adherence to BP self-management guidelines. The significance of the contribution would be high.

Weaknesses

- It is stated that “more intensive behavioral interventions…with wireless devices are needed” based on the lack of significant findings in the Ogedegbe study. Too little information is known about this study to draw this conclusion and support the proposed approach.

2. Investigator(s):

Strengths

- The PI is an early stage investigator with the necessary expertise in a relevant area. She was supported to develop and evaluate BPMED, an automated text message reminder system to improve medication adherence among 58 African Americans with uncontrolled HTN. The proposed study builds logically on her previous research.
- The team has representation from the disciplines of medicine, biostatistics, and social science, and has a history of working together to develop, implement, and evaluate self-monitoring interventions for behavior change.
- The team has collected supportive pilot data demonstrating the ability to recruit the targeted population, the ability to develop and test an accepted mHealth program, and improve medication adherence with an automated SMS reminder system.

Weaknesses

- None noted.

3. Innovation:

Strengths

- The use of commercially available devices is viewed as a strength.
- The targeted population is considered a strength.
- The recruitment method is unique and innovative.
- Focusing on multiple health behaviors is a strength.
- The objective measure of medication adherence is a strength.

Weaknesses

- None noted.

4. Approach:

Strengths

- The behavioral framework is clearly described and applies to the proposed study aims.
- The objective measure of medication adherence is a strength.
- The recruitment approach is well justified and planned.
- Statistical approach is sound.

Weaknesses

- The argument that the mHealth approach will be superior to the paper approach because “cell phones are already integrated into routine daily life and thus more accessible” is questionable. This assumes the major barrier for self-management amongst the target population is a lack of access to self-monitoring tools. Is there any evidence to support this claim? Any lessons learned from the BPMED study? Paper logs are also highly accessible. Further, there is evidence that physically writing information down facilitates better processing of information and better mastery. Skills mastery is a key component of the IMB model which the program is based upon. Thus it could be argued that the paper log system might actually result in better outcomes. It remains unclear why it is believe the mHealth approach will result in better self-monitoring than the paper log approach.
- Details regarding the “individually tailor in-app messages” are lacking. What will these message focus on?
- The use of the BRFSS measure is not considered a strong measurement tool for evaluating the efficacy of individual level interventions as the questions are broad.
- The BRFSS for measuring physical activity is a weakness. This is not an ideal self-report measure for evaluating the efficacy of individual level interventions.

5. Environment:

Strengths

- Laboratory and clinical facilities at the Detroit Medical Center, Wayne State University and Vanderbilt University are strong.
- Scientific environment at University of Michigan is very strong.
- There are many examples of a strong university commitment to the success of the PI.
- The Vibrent group adds a level of expertise for developing the mHealth tool.

Weaknesses

- None noted.

Protections for Human Subjects:

Acceptable Risks and/or Adequate Protections

Data and Safety Monitoring Plan (Applicable for Clinical Trials Only):

Acceptable

Inclusion of Women, Minorities and Children:

- Sex/Gender: Distribution justified scientifically
- Race/Ethnicity: Distribution justified scientifically
- Inclusion/Exclusion of Children under 21: Excluding ages < 21 justified scientifically

Vertebrate Animals:

Not Applicable (No Vertebrate Animals)

Biohazards:

Not Applicable (No Biohazards)

Revision:

- The investigative team was very responsive to the previous comments made by reviewers and should be commended.

Budget and Period of Support:

Recommend as Requested

THE FOLLOWING SECTIONS WERE PREPARED BY THE SCIENTIFIC REVIEW OFFICER TO SUMMARIZE THE OUTCOME OF DISCUSSIONS OF THE REVIEW COMMITTEE, OR REVIEWERS' WRITTEN CRITIQUES, ON THE FOLLOWING ISSUES:

PROTECTION OF HUMAN SUBJECTS (Resume): ACCEPTABLE

INCLUSION OF WOMEN PLAN (Resume): ACCEPTABLE

INCLUSION OF MINORITIES PLAN (Resume): ACCEPTABLE

INCLUSION OF CHILDREN PLAN (Resume): ACCEPTABLE

COMMITTEE BUDGET RECOMMENDATIONS: The budget was recommended as requested.

NIH has modified its policy regarding the receipt of resubmissions (amended applications). See Guide Notice NOT-OD-14-074 at http://grants.nih.gov/grants/guide/notice-files/NOT-OD-14-074.html. The impact/priority score is calculated after discussion of an application by averaging the overall scores (1-9) given by all voting reviewers on the committee and multiplying by 10. The criterion scores are submitted prior to the meeting by the individual reviewers assigned to an application, and are not discussed specifically at the review meeting or calculated into the overall impact score. Some applications also receive a percentile ranking. For details on the review process, see http://grants.nih.gov/grants/peer_review_process.htm#scoring.

MEETING ROSTER

Behavioral Medicine, Interventions and Outcomes Study Section
Risk, Prevention and Health Behavior Integrated Review Group
CENTER FOR SCIENTIFIC REVIEW
BMIO
October 05, 2015 - October 06, 2015

CHAIRPERSON

PALERMO, TONYA M, PHD
PROFESSOR
DEPARTMENT OF ANESTHESIOLOGY
AND PAIN MEDICINE SEATTLE
CHILDREN'S HOSPITAL RESEARCH INSTITUTE
UNIVERSITY OF WASHINGTON
SEATTLE, WA 98145

MEMBERS

AIKENS, JAMES E, PHD
ASSOCIATE PROFESSOR
DEPARTMENT OF FAMILY MEDICINE
UNIVERSITY OF MICHIGAN
ANN ARBOR, MI 48109

BARRETT, A M, MD *
DIRECTOR OF STROKE RESEARCH, KESSLER
FOUNDATION
PROFESSOR OF PHYSICAL MEDICINE
AND REHABILITATION
RUTGERS NEW JERSEY MEDICAL SCHOOL
WEST ORANGE , NJ 07052

BOND, DALE S, PHD
ASSOCIATE PROFESSOR
WEIGHT CONTROL
AND DIABETES RESEARCH CENTER
THE MIRIAM HOSPITAL
BROWN ALPERT MEDICAL SCHOOL
PROVIDENCE, RI 02903

BURNS, JOHN W, PHD *
PROFESSOR
DEPARTMENT OF BEHAVIORAL SCIENCES
RUSH UNIVERSITY MEDICAL CENTER
CHICAGO, IL 60612

CARR, LUCAS J, PHD *
ASSISTANT PROFESSOR
DEPARTMENT OF HEALTH
AND HUMAN PHYSIOLOGY
UNIVERSITY OF IOWA
IOWA CITY, IA 52242

DURANT, RAEGAN WINSTON, MD *
ASSOCIATE PROFESSOR
DIVISION OF PREVENTIVE MEDICINE
UNIVERSITY OF ALABAMA AT BIRMINGHAM
BIRMINGHAM, AL 35294

ESTABROOKS, PAUL , PHD *
PROFESSOR, DEPARTMENTS OF HUMAN NUTRITION,
FOODS AND EXERCISE
CENTER FOR TRANSLATIONAL OBESITY RESEARCH
VIRGINIA POLYTECHNIC INSTITUTE
AND STATE UNIVERSITY
ROANOKE, VA 24016

FEDELE, DAVID A, PHD *
ASSISTANT PROFESSOR
DEPARTMENT OF CLINICAL
AND HEALTH PSYCHOLOGY
UNIVERSITY OF FLORIDA
GAINESVILLE, FL 32610

GRIGSBY, JAMES P, PHD *
PROFESSOR
DEPARTMENTS OF MEDICINE
AND PSYCHOLOGY
UNIVERSITY OF COLORADO
DENVER, CO 80217

HOMMEL, KEVIN , PHD
ASSOCIATE PROFESSOR OF PEDIATRICS
CENTER FOR ADHERENCE
AND SELF-MANAGEMENT CINCINNATI
CHILDREN'S HOSPITAL MEDICAL CENTER
UNIVERSITY OF CINCINNATI
CINCINNATI, OH 45229

HOVELL, MELBOURNE F, PHD
DISTINGUISHED PROFESSOR AND DIRECTOR
CENTER FOR BEHAVIORAL EPIDEMIOLOGY
AND COMMUNITY HEALTH
SAN DIEGO STATE UNIVERSITY
SAN DIEGO, CA 92123

IM, EUN-OK , PHD *
PROFESSOR AND MAJORIE O RENDELL ENDOWED
PROFESSOR IN HEALTHY NURSING TRANSITIONS
SCHOOL OF NURSING
UNIVERSITY OF PENNSYLVANIA
PHILADELPHIA, PA 19104

KARP, JORDAN F, MD
ASSOCIATE PROFESSOR
DEPARTMENT OF PSYCHIATRY
UNIVERSITY OF PITTSBURGH
PITTSBURGH, PA 15213

KRULL, KEVIN R, PHD
PROFESSOR AND MEMBER
DEPARTMENT OF EPIDEMIOLOGY
AND CANCER CONTROL
ST. JUDE CHILDREN'S
RESEARCH HOSPITAL
MEMPHIS, TN 38105

LACKNER, JEFFREY M, PSYD *
PROFESSOR OF MEDICINE
BEHAVIORAL MEDICINE CLINIC
DEPARTMENT OF MEDICINE
UNIVERSITY AT BUFFALO MEDICAL SCHOOL
BUFFALO, NY 14215

MA, JUN , MD, PHD
PROFESSOR
HEALTH POLICY AND ADMINISTRATION
DEPARTMENT OF MEDICINE
SCHOOL OF PUBLIC HEALTH
UNIVERSITY OF ILLINOIS AT CHICAGO
CHICAGO, IL 60608

MILLER, SUZANNE M, PHD
PROFESSOR/DIRECTOR
DEPARTMENT OF PSYCHOSOCIAL
AND BEHAVIORAL MEDICINE
FOX CHASE CANCER CENTER
TEMPLE UNIVERSITY HEALTH SYSTEM
PHILADELPHIA, PA 19111

OLOMU, ADE B, MD, MS
PROFESSOR AND VICE CHAIR FOR RESEARCH
DEPARTMENT OF MEDICINE
MICHIGAN STATE UNIVERSITY
EAST LANSING, MI 48824

PARK, CRYSTAL L, PHD
PROFESSOR
DEPARTMENT OF PSYCHOLOGY
UNIVERSITY OF CONNECTICUT
STORRS, CT 06269

PENEDO, FRANK J, PHD
PROFESSOR
DEPARTMENT OF MEDICAL
AND SOCIAL SCIENCES
FEINBERG SCHOOL OF MEDICINE
NORTHWESTERN UNIVERSITY
CHICAGO, IL 60611

PRIGERSON, HOLLY G, PHD *
DIRECTOR
CENTER FOR RESEARCH ON END OF LIFE CARE
IRVING SHERWOOD WRIGHT
PROFESSOR OF MEDICINE
WEILL CORNELL MEDICAL COLLEGE â€ƒ
NEW YORK CITY, NY 10065

RAVENELL, JOSEPH E, MD
ASSISTANT PROFESSOR
DEPARTMENT OF POPULATION HEALTH
SCHOOL OF MEDICINE
NEW YORK UNIVERSITY
NEW YORK , NY 10016

RODRIGUE, JAMES R, PHD
PROFESSOR AND VICE CHAIRMAN
DEPARTMENT OF SURGERY
BETH ISRAEL DEACONESS
MEDICAL CENTER
BOSTON, MA 02215

STEWART, JESSE C, PHD *
ASSOCIATE PROFESSOR
DEPARTMENT OF PSYCHOLOGY
INDIANA UNIVERSITY
PURDUE UNIVERSITY INDIANAPOLIS
INDIANAPOLIS, IN 46202

STREISAND, RANDI , PHD *
ASSOCIATE PROFESSOR
DIRECTOR OF PSYCHOLOGY
RESEARCH
CHILDREN'S NATIONAL MEDICAL CENTER
WASHINGTON, DC 20010

WEINGER, KATIE , EDD
ASSOCIATE PROFESSOR OF PSYCHIATRY
INVESTIGATOR
JOSLIN DIABETES CENTER
HARVARD UNIVERSITY
BOSTON, MA 02215

SCIENTIFIC REVIEW OFFICER

MANN, LEE S, PHD
SCIENTIFIC REVIEW OFFICER
CENTER FOR SCIENTIFIC REVIEW
NATIONAL INSTITUTES OF HEALTH
BETHESDA, MD 20892

EXTRAMURAL SUPPORT ASSISTANT

JORDAN, CAROLYN
CENTER FOR SCIENTIFIC REVIEW
NATIONAL INSTITUTES OF HEALTH
BETHESDA, MD 20892

* Temporary Member. For grant applications, temporary members may participate in the entire meeting or may review only selected applications as needed.

Consultants are required to absent themselves from the room during the review of any application if their presence would constitute or appear to constitute a conflict of interest.
